# Image quality of 4D in-treatment CBCT acquired during lung SBRT using FFF beam: a phantom study

**DOI:** 10.1186/s13014-020-01668-3

**Published:** 2020-09-25

**Authors:** Jihun Kim, Ki Chang Keum, Ho Lee, Chae-Seon Hong, Kwangwoo Park, Jin Sung Kim

**Affiliations:** Department of Radiation Oncology, Yonsei University College of Medicine, 50-1 Yonsei-ro, Seodaemoon-gu, Seoul, South Korea

**Keywords:** Image quality, In-treatment, Cone-beam computed tomography, Stereotactic body radiation therapy, Lung, Flattening filter free

## Abstract

**Background:**

Rotational beam delivery enables concurrent acquisition of cone-beam CT (CBCT), thereby facilitating further geometric verification of patient setup during radiation treatment. However, it is challenging to acquire CBCT during stereotactic body radiation therapy (SBRT) using flattening-filter free X-ray beams, in which a high radiation dose is delivered. This study presents quantitative evaluation results of the image quality in four-dimensional (4D) in-treatment CBCT acquired during SBRT delivery.

**Methods:**

The impact of megavoltage (MV) scatter and acquisition parameters on the image quality was evaluated using Catphan 503 and XSight lung tracking phantoms. The in-treatment CBCT images of the phantoms were acquired while delivering 16 SBRT plans. The uniformity, contrast, and contrast-to-noise ratio (CNR) of the in-treatment CBCT images were calculated and compared to those of CBCT images acquired without SBRT delivery. Furthermore, the localizing accuracy of the moving target in the XSight lung phantom was evaluated for 10 respiratory phases.

**Results:**

The CNR of the 3D-reconstucted Catphan CBCT images was reduced from 6.3 to 2.6 due to the effect of MV treatment scatter. Both for the Catphan and XSight phantoms, the CBCT image quality was affected by the tube current and monitor units (MUs) of the treatment plan. The lung target in the XSight tracking phantom was most visible for extreme phases; the mean CNRs of the lung target in the in-treatment CBCT images (with 40 mA tube current) across the SBRT plans were 3.2 for the end-of-exhalation phase and 3.0 for the end-of-inhalation phase. The lung target was localized with sub-millimeter accuracy for the extreme respiratory phases.

**Conclusions:**

Full-arc acquisition with an increased tube current (e.g. 40 mA) is recommended to compensate for degradation in the CBCT image quality due to unflattened MV beam scatter. Acquiring in-treatment CBCT with a high-MU treatment beam is also suggested to improve the resulting CBCT image quality.

## Background

For lung stereotactic body radiation therapy (SBRT), appropriate management of respiratory motion is critical for accurate delivery of radiation beams to the target. Respiratory motion of lung target has been managed by various techniques, such as motion encompassing, respiratory gating, breath-hold, and forced shallow breathing with abdominal compression as described in the report of the American Association of Physicists in Medicine Task Group 76 [1].

Monitoring during-treatment position of the lung target is essential for all motion management techniques. For instance, for respiratory gating techniques, beam delivery is triggered by gating signals, either amplitude-based or phase-based, obtained using external surrogate markers [2–4] or implanted fiducial markers [5–7]. Furthermore, for motion-encompassing techniques, although the internal target volume is defined to cover the entire range of the lung target motion due to the patient’s respiration, it is still necessary to verify that the lung target moves within the pre-defined range estimated using four-dimensional (4D) respiratory-correlated CT. While fluoroscopic image-based target monitoring is a well-established method for CyberKnife, which is a robotic radiosurgery system equipped with an orthogonal pair of x-ray imaging devices, it has been recently investigated for C-arm linac machines [8–10].

To monitor intrafraction position of the radiation target during volumetric-modulated arc therapy (VMAT)-based lung SBRT, kV projections have been obtained during VMAT delivery and reconstructed to 3D or 4D cone-beam CT (hereinafter, referred to as in-treatment CBCT). The clinical feasibility of acquiring CBCT during VMAT delivery was evaluated by initial studies [11–14]. Furthermore, 4D in-treatment CBCT was clinically implemented to evaluate lung target coverage across all respiratory phases during VMAT-based SBRT [15, 16].

The image quality of in-treatment CBCT has been evaluated in recent studies [17, 18]. Shimohigashi et al. investigated the impact of prescription dose (dose rate or gantry speed) on the image quality of 4D in-treatment CBCT images [18]. However, in the previous studies, in-treatment CBCT images were acquired only under relatively moderate dose rates without considering flattening filter-free (FFF) X-ray beams; FFF X-ray beams have been frequently used for lung SBRT due to their high dose rate, thereby reducing the total beam-on time. It is an interesting research question how visible the lung target is on 4D in-treatment CBCT acquired with concurrent delivery of such highly intensive radiation beams.

To the best of the author’s knowledge, this study is the first to evaluate the image quality of 4D in-treatment CBCT acquired during lung SBRT using FFF x-ray beams. Phantom studies were performed (1) to quantitatively evaluate the megavoltage (MV) treatment beam scatter, (2) to investigate the impact of the acquisition parameters (tube current, acquisition angle, MV treatment beam energy, and plan-specific parameter (e.g. monitor unit (MU)) on the image quality, and (3) to evaluate the visibility and localization of the lung target for each respiratory phase using 4D in-treatment CBCT.

## Methods

### Overview

**Fig. 1:**
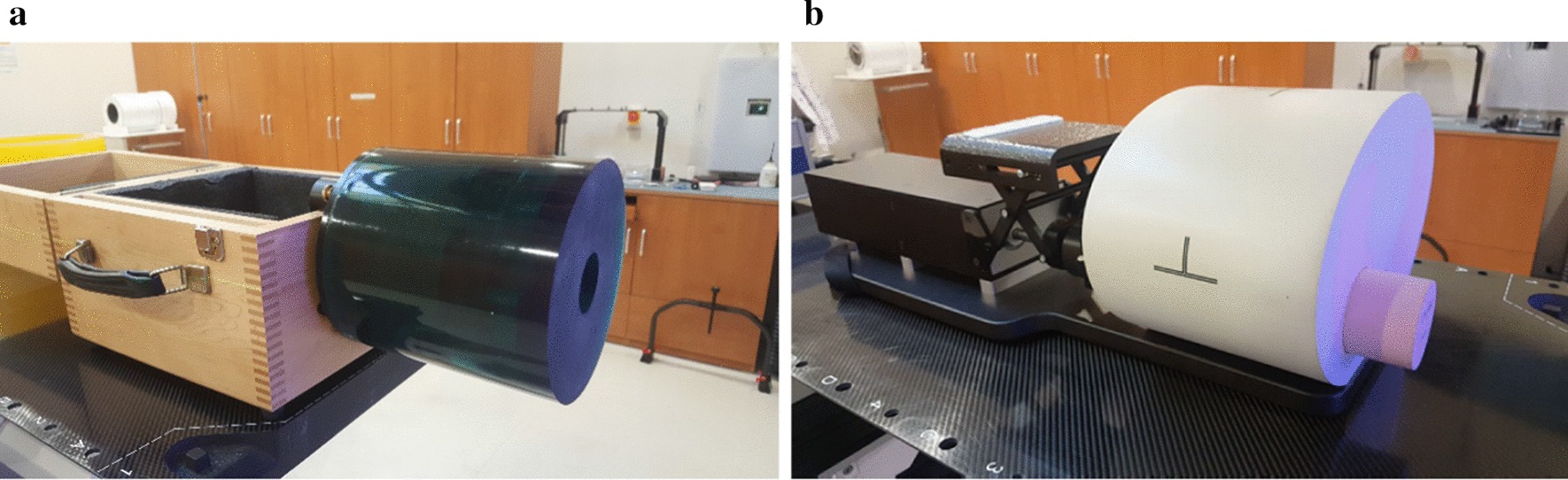
Phantoms used to evaluate cone-beam CT image quality: **a** Catphan 503 phantom and **b** XSight lung tracking phantom

Phantom studies were performed to evaluate the image quality of 4D in-treatment CBCT. The following aspects were mainly investigated in this study: (1) the impact of the MV beam scatter on the CBCT image quality, (2) the impact of the acquisition parameters on the CBCT image quality, (3) the accuracy of localizing the lung target on the respiratory phases. Two phantoms were used for these investigations (see Fig. 1): a Catphan 503 phantom (The Phantom Laboratory, Salem, NY, USA) and an XSight lung tracking phantom (CIRS Inc., Norfolk, VA, USA). It is noted that the study of using the Catphan phantom was performed without a motion platform and the CBCT image quality was evaluated on 3D-reconstructed images. As preliminary steps towards the evaluation of 4D in-treatment CBCT images of the XSight lung tracking phantom, the impact the acquisition parameters as well as the impact of the MV scatter was evaluated using the 3D Catphan CBCT images. The impact of the acquisition parameters and localization accuracy/visibility of the lung target were investigated on the 4D XSight tracking phantom.

### Impact of MV treatment beam scatter

In order to quantitatively evaluate the impact of the MV treatment beam scatter on the CBCT image quality, CBCT scans of the Catphan 503 phantom were acquired with four different imaging protocols as summarized in Table 1 using a gantry-mounted kV imager (XVI, Elekta Oncology Systems). The pretreatment CBCT imaging protocols used in this investigation are currently clinically used to examine patients with a lung target. It should be noted that, for the sake of simplicity, CBCT acquired without MV treatment beam delivery is denoted by pretreatment CBCT since, in general, it is acquired prior to treatment for patient setup. As an initial test, the in-treatment CBCT imaging protocols in Table 1 were created by simply adding the parameters specifically required for in-treatment CBCT acquisition without adjusting the other acquisition parameters, such as the tube voltage, tube current, exposure time, and rotation angle.

**Table 1:** Summary of four cone-beam CT imaging protocols used in this study

	3D		4D	
	Pretreatment	In-treatment	Pretreatment	In-treatment
Image acquisition parameters				
MV treatment beam	No	Yes	No	Yes
Tube voltage (kV)	120	120	120	120
Tube current (mA)	40	40	20	20
Exposure time (ms)	40	40	16	16
Collimator	M20	M20	S20	S20
Rotation angle (°)	360	360	200	200
Gantry speed (°/min)	360	–	67	–
Acquisition interval (°)	–	0.1	–	0.1
Estimated time (s)^a^	60	99	180	55
Analysis results				
#projections acquired	333	512	1010	266
Contrast (%)	9.6	10.1	8.0	5.5
Contrast-to-noise ratio	10.0	9.5	10.7	2.6
Overall uniformity (%)	98.5	98.0	97.1	89.1
Minimum uniformity (%)	98.8	98.4	98.4	95.7

The CBCT acquired without MV treatment beam delivery is denoted by pretreatment CBCT as it is acquired prior to treatment. The results of an initial image quality test for the listed protocols are summarized

^a^The time for the in-treatment CBCT images was estimated as the beam-on time expected to deliver the VMAT plan for patient 1. In general, the time to acquire an in-treatment CBCT image varies depending on the VMAT plan, which is concurrently delivered

For the intrafraction CBCT acquisition, a VMAT plan for a lung cancer patient (patient 1) was delivered to the Catphan phantom. A two-arc VMAT plan was created for the lung SBRT using FFF 10 MV X-ray beams. Among the two arcs, the first arc plan, which was delivered during the CBCT acquisition, was created to deliver 2585.3 MU in 77 s along a full-arc gantry rotation (from − 179° to 179°). The corresponding gantry speed to deliver the first arc of the VMAT plan was estimated to be approximately 279°/min on average. The average planned dose rate was estimated to be 2014.5 MU/min.

For the CBCT acquisitions, two-dimensional projections were acquired and then reconstructed to multiple-phase three-dimensional image volumes. Prior to the CBCT reconstruction, the acquired projections were sorted into ten respiratory phases (0, 10, 20, …, 90) by an automatic phase-sorting algorithm [19] in XVI. Among the ten respiratory phases, 0-phase and 50-phase represent end-of-exhalation and end-of-inhalation phases, respectively, and the other eight phases represent intermediate phases between the end respiratory phases.

It is noteworthy to mention that different numbers of projections were acquired for the pretreatment and in-treatment CBCT protocols due to the concurrent delivery of MV treatment beams for the in-treatment CBCT as summarized in Table 1. For the in-treatment CBCT acquisition, it is not necessary to define the gantry speed since the linac gantry rotates with time-varying angular speeds, which are optimized for radiotherapy treatment plan. Consequently, the number of projections acquired for the in-treatment CBCT could vary depending on the VMAT plans delivered with the CBCT acquisition. Instead of defining the gantry speed, an angular interval, so called the acquisition interval in XVI, was defined for acquiring the in-treatment CBCT; the acquisition interval is an acquisition parameter that defines the minimum gantry rotation, upon which kV projection acquisition is triggered (0.1° for this study as suggested in a previous study [20]). As summarized in Table 1, more projections were acquired for the three-dimensional (3D) in-treatment CBCT than the 3D pretreatment CBCT: 495 vs. 333 projections. On the other hand, a larger number of projections was acquired for the 4D pretreatment CBCT (1010) than for the 4D in-treatment CBCT (266) as gantry rotated more slowly for 4D pretreatment CBCT. Since the CBCT image quality is largely affected by the number of projections acquired [21–23], it was not feasible to evaluate the effect of the MV scatter from the results in Table 1.

In order to more appropriately evaluate the impact of the MV scatter on the CBCT image quality, the gantry speed of the 3D and 4D pretreatment CBCT imaging protocols was adjusted. For the 3D pretreatment CBCT acquisition, the gantry speed was reduced from 360 to 240°/min. For the 4D pretreatment CBCT acquisition, the gantry speed was adjusted from 67 to 254°/min. By adjusting the gantry speed, similar numbers of projections were acquired for the pretreatment and in-treatment CBCT protocols, leading to a fair comparison of the image quality between the pretreatment and in-treatment CBCTs. It should be noted that the aforementioned adjustments of the gantry speed, which largely affected the resulting CBCT image quality, were performed only for evaluating the MV scatter effect. For the other experiments, pretreatment CBCT images were acquired with the standard imaging protocols summarized in Table 1. The kV projections acquired with the 3D and 4D imaging protocols were reconstructed into 3D CBCT images.

**Fig. 2:**
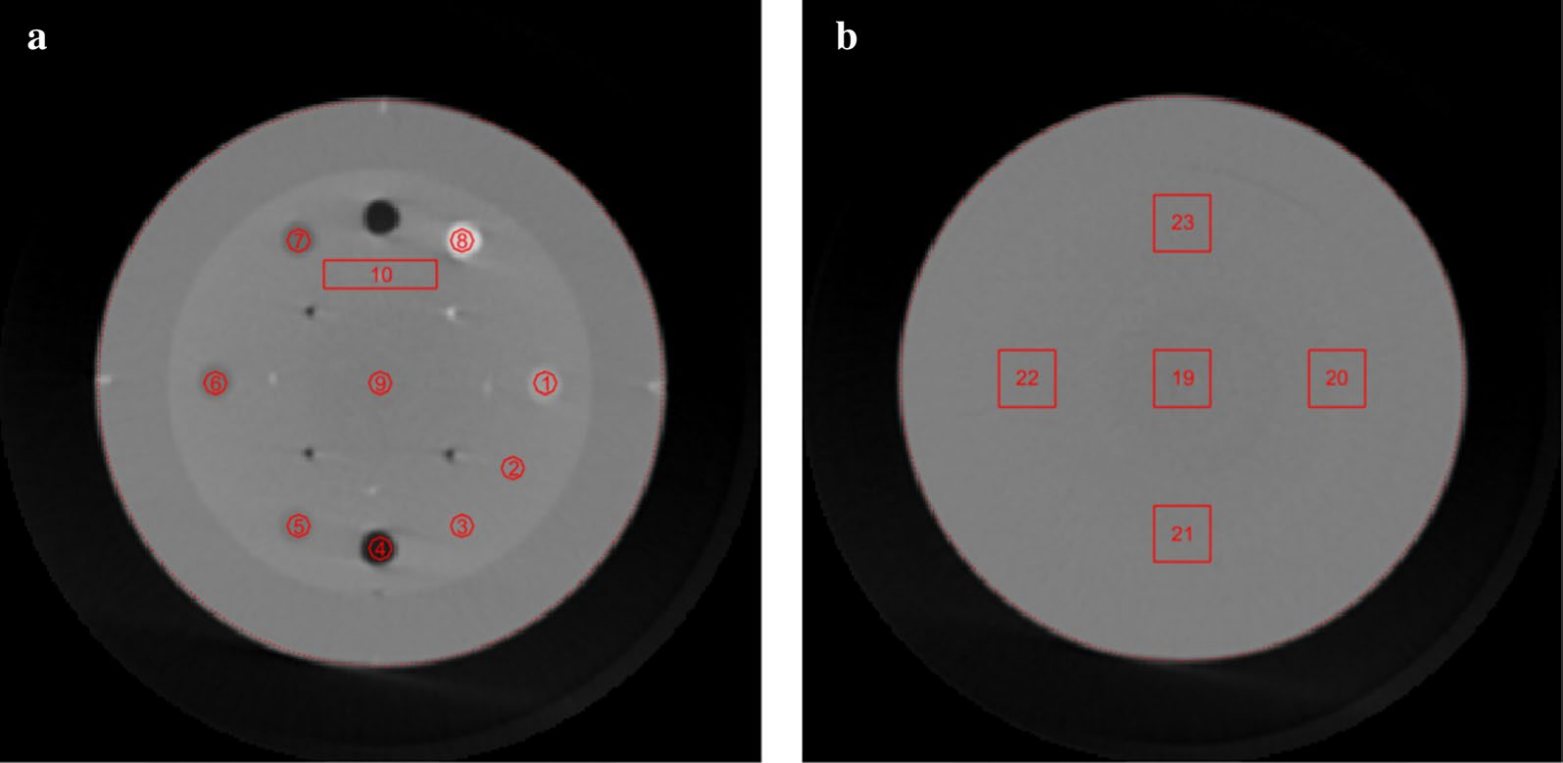
Axial cuts of two modules in the Catphan 503 phantom visualized with regions of interest used for quantitative evaluations of CBCT image quality. The two modules, **a** CTP404 and **b** CTP486, were used to calculate the contrast- and uniformity-related metrics, respectively

To quantitatively evaluate the image quality of the 3D-reconstructed CBCT images, several image quality metrics were calculated using DoseLab Pro 7.0 (Varian Medical Systems, Palo Alto, CA, USA), an image analysis software package. As illustrated in Fig. 2, the Catphan 503 phantom consisted of three modules, each of which was used to analyze different image quality metrics: the contrast and contrast-to-noise ratio (CNR) (CTP404 in Fig. 2a), and the overall and minimum uniformity (CTP486 in Fig. 2b). First, the CTP404 module has multiple inserts with various electron density values; 10 regions of interest (ROIs) detected by DoseLab are displayed in Fig. 2a. For this investigation, ROIs 1 (water-equivalent) and 2 (Delrin) were used to calculate contrast and CNR as follows:

$$Contrast=\frac{{S}_{2}-{S}_{1}}{{S}_{1}+{S}_{2}}\times 100\, (\%)$$

$${CNR}_{Catphan}=\frac{{S}_{2}-{S}_{1}}{{S}_{1}+{S}_{2}}/\frac{\sqrt{{\sigma }_{1}^{2}+{\sigma }_{2}^{2}}}{\sqrt{{S}_{1}^{2}+{S}_{2}^{2}}}$$

where *S*_1_ and *S*_2_ represents the mean pixel values of ROIs 1 and 2, respectively, whereas *σ*_1_ and *σ*_2_ represents the standard deviations of the pixel values in ROIs 1 and 2, respectively. Second, overall and minimum uniformities were calculated for ROIs 19–23 using an equation that calculates the uniformity metric for a region:

$$Uniformity=\left(1-\frac{{S}_{90}-{S}_{10}}{{S}_{90}+{S}_{10}}\right)\times 100\, (\%)$$

where *S*_90_ and *S*_10_ represent the 90th and 10th percentile of the pixel values in an ROI, respectively. The overall uniformity was calculated as the uniformity calculated for all of the ROIs. The minimum uniformity was calculated as the lowest value among the uniformity values calculated for each of ROIs 19–23.

### Impact of tube current, acquisition angle, and treatment beam energy

The impact of several important image acquisition parameters, such as the kV tube current, acquisition angle, and treatment beam energy, on the CBCT image quality was evaluated. Since image quality degradation was expected due to the MV scatter, the tube current was increased from 20 mA (vendor-recommended value for 4D pretreatment CBCT) to 25, 32, 40, and 50 mA. The 4D pretreatment protocol was the vendor-recommended CBCT imaging protocol for the patient thorax, known as the Symmetry protocol, as described in Table 1. In the Symmetry protocol, the gantry rotation is limited to 200°. In this investigation, for the 4D in-treatment CBCT acquisition, the Symmetry protocol was modified so that the CBCT projections were acquired during a full-arc rotation. The rationales behind acquiring 4D in-treatment CBCT with a full-arc rotation can be explained as follows: (1) full-arc acquisition can maximally cover the angular range of VMAT treatment beams, and (2) a larger number of projections can be acquired with a full-arc acquisition, leading to improved image quality. In order to investigate the impact of the treatment beam energy, for patient 1, another VMAT plan using 6 MV FFF was created so that the resulting dose distribution was similar to that with 10 MV FFF. The 6 MV FFF VMAT plan delivered 2805.2 MU in 99 s (estimated) for the first arc delivery; this case was referred to as patient 2 for simplicity of explanation. The CBCT image quality metrics defined above were calculated for all 3D-reconstructed CBCT images.

### CBCT acquisitions with various VMAT plans

It was investigated how the in-treatment CBCT image quality varies depending on the VMAT plan concurrently delivered with the CBCT acquisition. While each of the first-arc beams of 16 patient VMAT plans, which were created to treat lung cancer patients, was delivered to the Catphan phantom, kV projections were acquired with tube currents of 20 and 40 mA. The acquired projections were reconstructed into 3D CBCT images for the image quality evaluation. The MUs delivered during the arc delivery differed depending on the VMAT plan (prescription dose per fraction, number of arcs): 2062.6 ± 1221.2 MU (153.8 to 4524.9 MU); the fractional dose ranged from 8 to 12 Gy. Among the 16 VMAT plans, 11 plans were created using 6 MV FFF X-ray beams and 5 plans were created using 10 MV FFF x-ray beams. The quantitative image quality metrics explained above were calculated using DoseLab. The relationships between the CBCT image quality metrics and MUs (or number of projections acquired) were examined by calculating the Pearson correlation coefficient using MATLAB (MathWorks, Natick, MA, USA).

### Visibility and localization accuracy of lung target

An XSight lung tracking phantom was used to evaluate the visibility and localization accuracy of the lung target using 4D in-treatment CBCT. The lung tracking phantom consists of several anatomic regions with different physical densities, such as bony elements, soft tissue, and lungs, mimicking the patient thorax. A soft tissue target, which is a sphere with a diameter of 2.5 cm, was designed to move in the superior-inferior direction with a breathing cycle of 5 s and an excursion of 29.5 mm. The accuracy of localizing this moving target was evaluated for each of 10 respiratory phases by comparing the superior-inferior coordinates of the target center obtained using the 4D in-treatment CBCT images to those obtained using a 4D pretreatment CBCT image. The 16 VMAT plans were delivered during the in-treatment kV projection acquisition with tube currents of 20 and 40 mA. The kV projections were reconstructed into ten-phase 4D in-treatment CBCT images by an automatic phase-sorting algorithm [19], which is designed to detect dominant positional changes across projections.

**Fig. 3:**
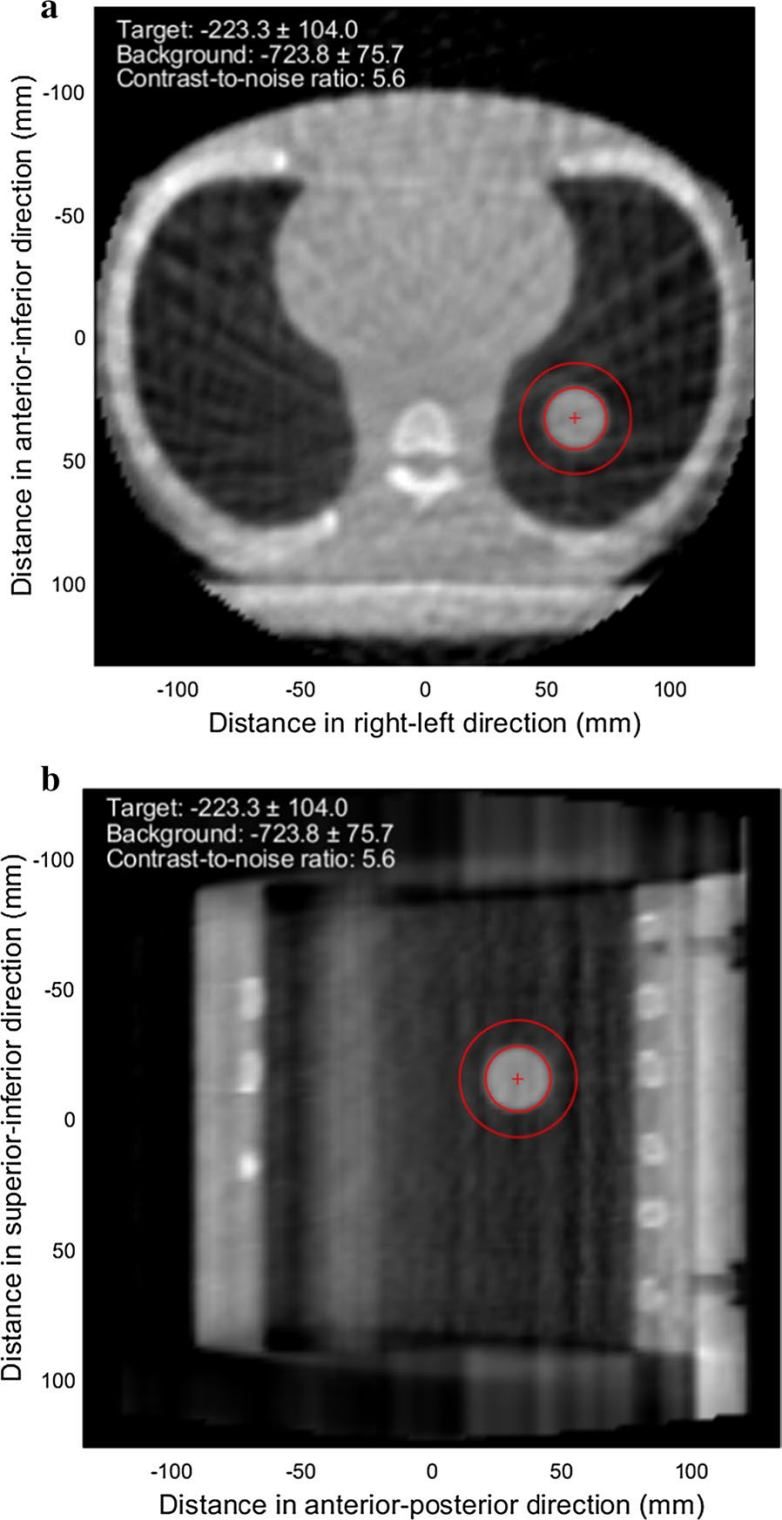
Illustration of the contrast-to-noise ratio (CNR) calculation for the spherical moving target in the XSight lung tracking phantom. On the **a** axial and **b** sagittal cuts that intersect with the target center, two circles are displayed, which represent the boundaries of the target (sphere) and background (spherical shell). The resulting CNR was 5.6 for the 0-phase (end of exhalation) pretreatment CBCT image

Furthermore, the CNR for the in-treatment 4D CBCT data sets of the XSight lung tracking phantom was calculated by locating a spherical volume with a diameter of 2.5 cm at the center of the lung target, which was obtained for the 4D pretreatment CBCT image as described in Fig. 3. The background HU value was calculated by defining a spherical shell with 1.0 cm thickness surrounding the spherical target structure. The CNR for the lung target in the XSight lung tracking phantom was calculated as follows:

$${CNR}_{XSight}=({S}_{\mathrm{T}}-{S}_{\mathrm{B}})/\frac{{(\sigma }_{\mathrm{T}}{+\sigma }_{\mathrm{B}})}{2}$$

where *S*_T_ and *S*_B_ represent the mean HU values for the target and background, respectively, whereas the standard deviations of the HU values for the target and background are denoted by *σ*_T_ and *σ*_B_, respectively. The CNR calculated for the XSight lung tracking phantom estimates the differentiability of the solid lung target from the surrounding materials (lung) compared to their noise level. An example of the CNR calculation was presented in Fig. 3: the resulting CNR was for the 0-phase pretreatment CBCT image was 5.6, indicating that the mean signal difference between the target and background was 5.6 times greater than the mean noise level of the two regions. A correlation analysis was performed to calculate the Pearson correlation coefficient between the CNR and MUs. A paired two-tailed t-test was conducted to evaluate a hypothesis that the CNRs between the 20 and 40 mA tube currents were statistically different. The statistical significance in the CNR differences was evaluated with a 95% confidence using MATLAB. The CNR data were approximately normally distributed, not violating the assumption for the t-test.

## Results

### Impact of MV treatment beam scatter

Table 2 compares the image quality of the 3D-reconstructed Catphan CBCT images acquired with the four different protocols. The uniformity measures slightly decreased due to the MV treatment beam scatter both for the 3D and 4D CBCT imaging protocols, indicating an increase in noise. Furthermore, the CNR decreased from 14.9 to 9.5 for the 3D CBCT protocol and from 6.3 to 2.6 for the 4D CBCT protocol due to a decrease in contrast and an increase in noise level resulting from the MV scatter. The impact of the number of projections on the CBCT image quality was successfully suppressed as can be observed from the fact that similar numbers of projections were obtained for the pretreatment and in-treatment CBCT images.

**Table 2:** Summary of four cone-beam CT imaging protocols with gantry speed adjusted for the pretreatment CBCT scans and corresponding image quality evaluation results

	3D		4D	
	Pretreatment	In-treatment	Pretreatment	In-treatment
Image acquisition parameters				
MV treatment beam	No	Yes	No	Yes
Tube voltage (kV)	120	120	120	120
Tube current (mA)	40	40	20	20
Exposure time (ms)	40	40	16	16
Collimator	M20	M20	S20	S20
Rotation angle (°)	360	360	200	200
Gantry speed (°/min)	240	–	254	–
Acquisition interval (°)	–	0.1	–	0.1
Estimated time (s)^a^	90	99	47	55
Analysis results				
#projections acquired	502	512	276	266
Contrast (%)	9.5	10.1	7.9	5.5
Contrast-to-noise ratio	14.9	9.5	6.3	2.6
Overall uniformity (%)	98.7	98.0	96.4	89.1
Minimum uniformity (%)	99.1	98.4	98.0	95.7

^a^The time for the in-treatment CBCT images was estimated for the case of delivering the VMAT plan for patient 1. In general, the time to acquire in-treatment CBCT images varies depending on the VMAT plan, which is concurrently delivered.

### Impact of tube current, acquisition angle, and treatment beam energy

**Fig. 4:**
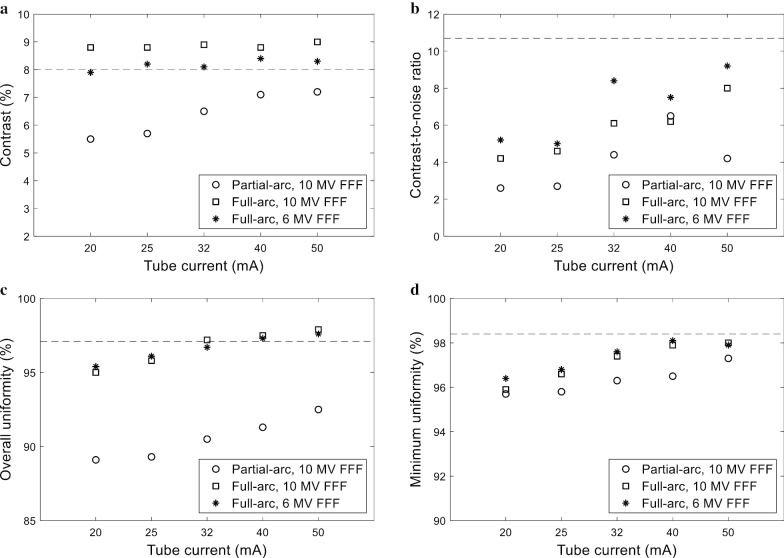
Quantitative metrics to evaluate the 3D-reconstructed in-treatment Catphan CBCT image quality plotted vs. the tube current: **a** contrast, **b** contrast-to-noise ratio, **c** overall uniformity, and **d** minimum uniformity. The in-treatment CBCT acquisition and image quality analysis were performed for different acquisition modes (partial-arc vs. full-arc) and different MV treatment beam energy values (6 vs. 10 MV FFF). The image quality metrics for the pretreatment CBCT acquired with the vendor-recommended Symmetry protocol are represented as dashed lines in each graph

The quality of the 3D-reconstructed Catphan CBCT images acquired with various tube currents (20, 25, 32, 40, and 50 mA), acquisition angles (200° and 360°), and treatment beam energies (6 and 10 MV FFF) was quantitatively analyzed and the results are summarized in Fig. 4. First, in general, as the tube current increased, the CBCT image quality was improved. Specifically, the CNR, overall uniformity, and minimum uniformity were improved as the tube current increased from 20 to 50 mA.

Second, a larger number of projections was acquired for the full-arc acquisition compared to the standard 200° acquisition. For the CBCT scans acquired with the 10 MV FFF VMAT plan delivery, the number of kV projections acquired was 270 ± 4 for the partial-arc (200°) acquisition compared to 541 ± 10 for the full-arc acquisition. All quantitative measures were improved by the full-arc acquisition. The mean CNRs for the partial-arc and full-arc acquisitions were 4.1 and 5.8, respectively (Fig. 4). The overall uniformity increased, on average, from 90.5 to 96.7 by increasing the acquisition angular range from 200° to 360°.

Third, when comparing the CBCT image quality between the in-treatment CBCT images acquired with the delivery of the 6 and 10 MV FFF VMAT plans, two major factors need to be considered: (1) the MV treatment beam scatter and (2) the total number of kV projections. A larger number of projections (619 ± 1) was acquired for the CBCT images acquired with the 6 MV FFF VMAT plan compared to those with the 10 MV FFF VMAT plan due to the lower dose (MU) rate (therefore, longer treatment time). The effect of the MV treatment beam scatter on the CBCT image quality is expected to be large for low treatment beam energy as reported in previous work [17]. As a result, the impact of the MV treatment beam energy differed for the contrast and CNR. The contrast was higher for the CBCT images acquired with the 10 MV FFF VMAT plan while the CNR was higher for those acquired with the 6 MV FFF VMAT plan, indicating a low level of noise, which was possibly due to the large number of projections. The MV treatment beam energy was found to have a negligible influence on the overall and minimum uniformities. Overall, the CBCT image quality was more affected by the acquisition angle than the MV treatment beam energy as illustrated in Fig. 4.

### CBCT acquisitions with various VMAT plans

**Fig. 5:**
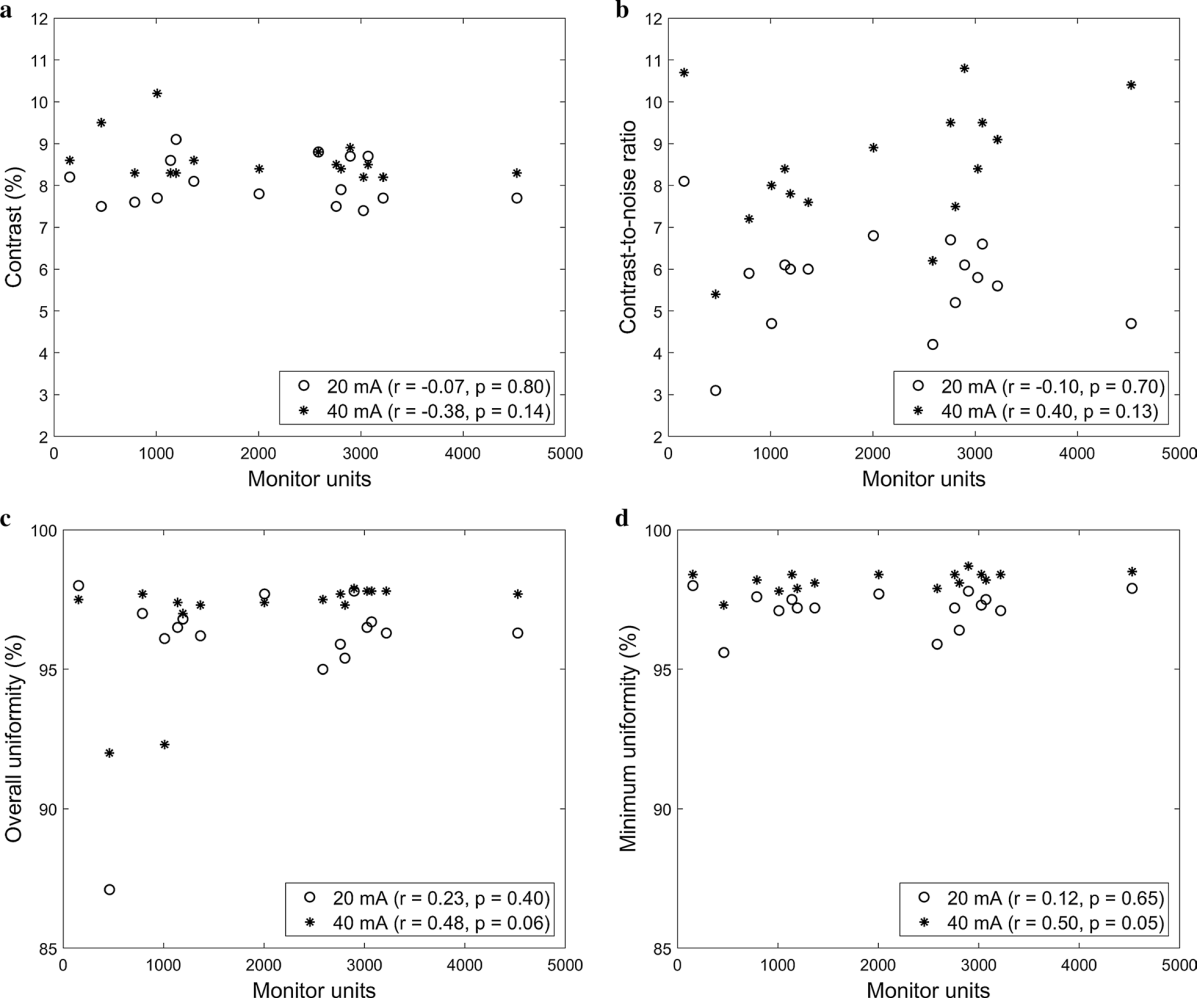
Scatter plots of the **a** contrast, **b** contrast-to-noise ratio, **c** overall uniformity, and **d** minimum uniformity calculated using the Catphan phantom against the monitor units. The CBCT image quality metrics were compared between the tube currents of 20 and 40 mA. The Pearson correlation coefficients *r* and corresponding p-values *p* are presented

Figure 5 presents the quantitative analysis results of the image quality for the 3D-reconstructed CBCT images of the Catphan phantom, obtained concurrently with the 16 VMAT plans. In Fig. 5, each of the image quality metrics is plotted against the MUs with the Pearson correlation coefficient *r* and corresponding p-value *p* (calculated for 95% statistical significance) presented.

The image quality metrics at a high tube current (40 mA) were more significantly affected by the VMAT plans (or MUs) than those at low tube current (20 mA) as can be observed from the Pearson correlation coefficients and *p*-values. The Pearson correlation coefficients were higher with smaller p-values for the CBCT images acquired with 40 mA than those acquired with 20 mA. For instance, the Pearson correlation coefficients for the CNR were − 0.10 (*p* = 0.70) for 20 mA vs. 0.40 (*p* = 0.13) for 40 mA. For the 40 mA tube current, when more MUs were delivered, a higher CNR resulted from the in-treatment CBCT. Similar (positive) correlations with the MUs were found for the overall and minimum uniformities. The contrast was found to be negatively (but weakly) correlated with the MUs. On the other hand, for the 20 mA tube current, no correlation was observed for any image quality metrics.

The statistics (mean, standard deviation, minimum, and maximum) of the image quality metrics across the 16 VMAT plans are summarized in Table 3. The number of kV projections acquired varied across the VMAT plans delivered with the CBCT acquisition: 617 ± 237 (346 to 1194) for the 40 mA tube current. A statistically significant correlation was observed between the MUs and number of projections acquired (*r* = 0.92, *p* = 0.00 for the 20 mA tube current, *r* = 0.94, *p* = 0.00 for the 40 mA tube current). In other words, when more MUs were delivered, more kV projections were acquired. Therefore, the relationships found between the number of projections acquired and CBCT image quality metrics were similar to those between the MUs and CBCT image quality metrics as shown in Fig. 5.

**Table 3:** Statistics (mean, standard deviation, and range) of the number of projections, monitor units, and CBCT image quality metrics calculated across 16 VMAT plans compared between the CBCT images acquired with the tube currents of 20 and 40 mA

	Tube current: 20 mA			Tube current: 40 mA		
	Mean, standard deviation (range)	Pearson correlation		Mean, standard deviation (range)	Pearson correlation	
		*r*	*p*		*r*	*p*
Number of projections acquired	623 ± 225 (379–1170)			617 ± 237 (346–1194)		
Monitor units	2063 ± 1221 (154–4525)	0.92	0.00	2063 ± 1221 (154–4525)	0.94	0.00
Contrast (%)	8.1 ± 0.5 (7.4–9.1)	− 0.10	0.72	8.6 ± 0.5 (8.2–10.2)	− 0.41	0.12
Contrast-to-noise ratio	5.7 ± 1.2 (3.1–8.1)	− 0.04	0.89	8.5 ± 1.5 (5.4–10.8)	0.55	0.03
Overall uniformity (%)	96.0 ± 2.5 (87.1–98.0)	0.32	0.23	96.9 ± 1.9 (92.0–97.9)	0.48	0.06
Minimum uniformity (%)	97.2 ± 0.7 (95.6–98.0)	0.31	0.24	98.2 ± 0.3 (97.3–98.7)	0.59	0.02

Furthermore, the Pearson correlation coefficients *r* and corresponding p-value *p* were calculated between the number of projections acquired and each of the other variables (the monitor units and CBCT image quality metrics)

Moreover, the impact of the tube current on the CBCT image quality can be observed from the image quality evaluation results presented in Table 3. The CNR, overall uniformity, and minimum uniformity increased as the tube current increased from 20 to 40 mA. The mean and standard deviation of the CNR, overall uniformity, and minimum uniformity values were 5.7% ± 1.2%, 96.0% ± 2.5%, and 97.2% ± 0.7%, respectively, for the 20 mA tube current vs. 8.5% ± 1.5%, 96.9% ± 1.9%, and 98.2% ± 0.3%, respectively, for the 40 mA tube current.

### Visibility and localization accuracy of lung target

It was not feasible to localize the lung target for several of the intermediate phases, in which the target moved rapidly. Therefore, the localization accuracy of the lung target was analyzed and reported only for the 0- and 50-phases, which corresponded to the end-of-exhalation and end-of-inhalation phases, respectively. The resulting coordinates were compared to those obtained with the pretreatment 4D CBCT images. The localization results are summarized in Table 4. The absolute localization error for the 0-phase was 0.5 ± 0.4 mm for the 20 mA tube current and 0.3 ± 0.2 mm for the 40 mA tube current. Sub-millimeter localization accuracy was also achieved for the 50-phase: 0.3 ± 0.3 mm for the 20 mA tube current and 0.1 ± 0.1 mm for the 40 mA tube current. Although a statistically significant correlation was found between the MU and 50-phase localization error for the CBCT images obtained with the 40 mA tube current, all of the values were quite small, ranging from − 0.3 to 0.1 mm. In general, the excursion estimated using in-treatment CBCT was smaller than the known motion magnitude (29.5 mm) for both tube currents; the excursion error was − 0.9 ± 0.5 mm for 20 mA and − 0.7 ± 0.2 mm for 40 mA, indicating potential underestimation of the lung target excursion when using the in-treatment CBCT.

**Table 4:** Statistics (absolute mean, standard deviation, and range) of the localization errors of the lung target in the XSight tracking phantom (0-phase and 50-phase) and excursion errors were compared between the 20 and 40 mA tube currents

	Tube current: 20 mA			Tube current: 40 mA		
	Absolute mean^a^, standard deviation (range)	Pearson correlation		Absolute mean, standard deviation (range)	Pearson correlation	
		*r*	*p*		*r*	*p*
Localization error (mm): 0-phase	0.5 ± 0.4 (− 0.6 to 1.0)	0.20	0.45	0.3 ± 0.2 (0.0 to 0.6)	0.36	0.16
Localization error (mm): 50-phase	0.3 ± 0.4 (− 1.2 to 0.2)	− 0.25	0.35	0.1 ± 0.1 (− 0.3 to 0.1)	0.62	0.01
Excursion error (mm)	− 0.9 ± 0.5 (− 1.6 to 0.2)	− 0.01	0.97	− 0.7 ± 0.2 (− 1.1 to 0.2)	− 0.45	0.08

The Pearson correlation coefficients *r* with the MUs and corresponding p-value *p* are also provided.

^a^Mean values are provided for the excursion error.

**Fig. 6:**
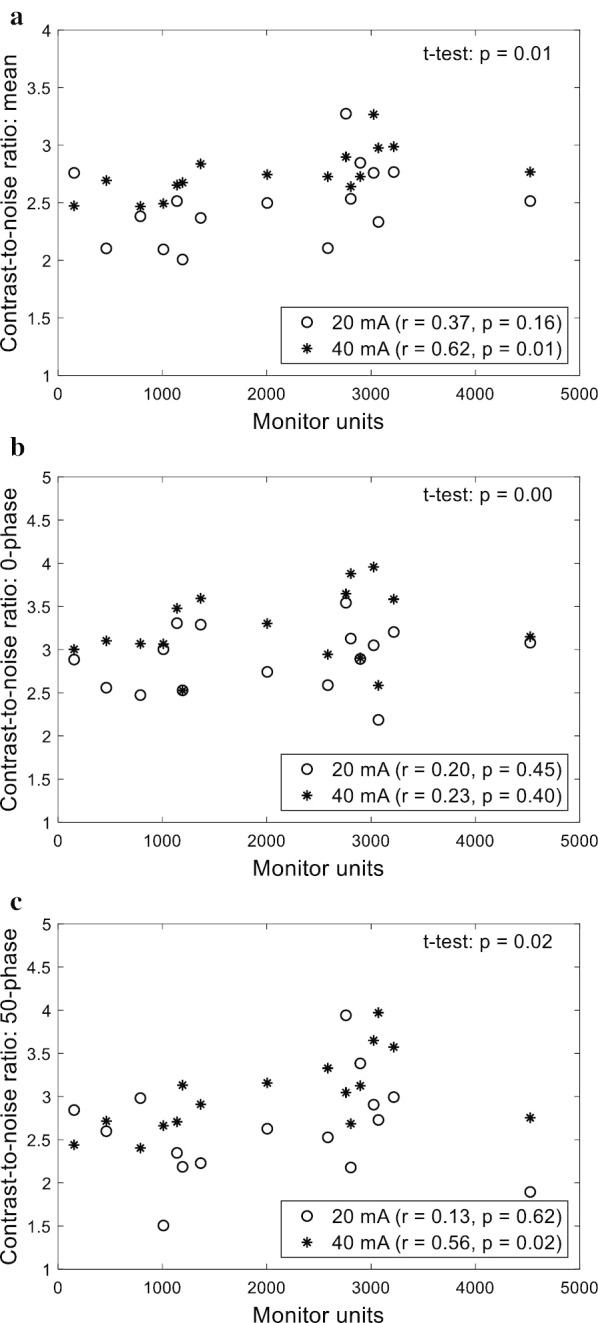
Scatter plots of the **a** mean CNR across the phases, **b** 0-phase CNR, and **c** 50-phase CNR against the monitor units. The CBCT image quality metrics were compared between the tube currents of 20 and 40 mA. The Pearson correlation coefficients and corresponding p-values are also presented

In Fig. 6, the CNRs calculated for 10 respiratory phases are plotted against the MUs of the VMAT plans concurrently delivered: (a) mean across the respiratory phases, (b) 0-phase, and (c) 50-phase. Statistically significant correlations were found for the mean and 50-phase CNRs with the 40 mA tube current; the Pearson correlation coefficients were 0.62 and 0.56, and the corresponding *p*-values were 0.01 and 0.02, respectively. On the other hand, weak correlations were found for the 0-phase CNR with the 40 mA tube current and all of the CNRs with the 20 mA tube current. As illustrated in Fig. 6, the p-values calculated by a paired t-test were 0.01 for the mean CNR, 0.00 for the 0-phase CNR, and 0.02 for the 50-phase CNR, indicating that the CNRs were significantly improved by increasing the tube current.

**Fig. 7:**
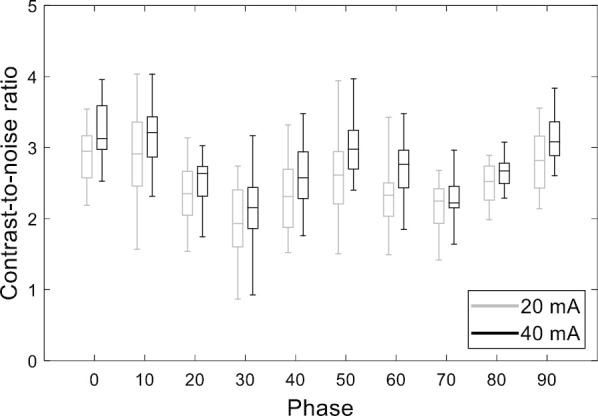
Box plots of the contrast-to-noise ratios calculated for the in-treatment 4D CBCT images obtained with 16 VMAT plans for 10 respiratory phases (0, 10, …, 90); 0 and 50 phases represent end-of-exhalation and end-of-inhalation phases, respectively, and the other phases represent intermediate phases

The CNRs calculated for the 16 VMAT plans for each of the 10 respiratory phases are presented as a box plot in Fig. 7. The CNR was higher for the extreme respiratory phases (exhalation and inhalation) than for the intermediate respiratory phases. An increase in the tube current resulted in an increase in the CNR for all respiratory phases.

## Discussion

This study presents quantitative evaluation results of the image quality of in-treatment CBCTs obtained with the delivery of high dose rate VMAT plans using FFF X-ray beams, which has not been investigated in previous studies. The effect of the MV treatment beam scatter on the in-treatment CBCT image quality was quantitatively analyzed. Moreover, the effects of several important factors, such as the tube current, acquisition angle range, and MU of the VMAT plan, on the CBCT image quality were investigated. The visibility of the lung target in the XSight tracking phantom, which was evaluated in terms of CNR, provides useful information on how visible the lung target is on 4D in-treatment CBCTs acquired during VMAT-based lung SBRT.

The CBCT image quality was found to be degraded by the scattered MV FFF X-ray beams concurrently delivered with the CBCT acquisition. Image quality degradation due to the MV scatter was reported in the previous studies on in-treatment CBCT obtained with unflattened X-ray beams [13, 17, 18, 24]. Shimohigashi et al. demonstrated that the in-treatment CBCT image quality was not significantly degraded when it was acquired with the delivery of VMAT plans using flattened X-ray beams [18]. However, in this previous study by Shimohigashi et al., the number of kV projections for the in-treatment CBCT was relatively large (979–1738) and the VMAT plans were delivered in a low dose rate using flattened X-ray beams. In contrast to the previous study, the in-treatment CBCT images were acquired in a SBRT setting, that is, with high dose rate VMAT delivery. For a fair comparison of the CBCT image quality between pretreatment and in-treatment CBCT images, the gantry speed for the pretreatment CBCT was adjusted, so that similar numbers of projections were obtained. All of the image quality metrics decreased due to the MV treatment beam scatter as indicated in Table 2. For instance, the CNR decreased from 6.3 to 2.6 due to the MV beam scatter for the 4D CBCT.

It was demonstrated that the image quality of in-treatment CBCT can be improved by increasing either the tube current or the angular range of the CBCT acquisition. The effect of the tube current on the CBCT image quality was evaluated using both the Catphan and XSight phantoms. As illustrated in Figs. 4 and 5, all of the image quality metrics calculated for the Catphan were improved by increasing the tube current. This positive effect of the tube current on the CBCT image equality was also demonstrated for the XSight lung tracking phantom. The CNR was improved by increasing the tube current from 20 to 40 mA, indicating an improvement in the lung target visibility. Furthermore, it was demonstrated that full-arc acquisition resulted in larger numbers of kV projections, thereby improving the quality of the reconstructed image. Based on the results of this investigation, the use of a tube current of 32–50 mA is recommended to compensate for the negative effect of the MV scatter.

Although the CBCT imaging dose was not measured in this investigation, reasonable estimations of the resulting imaging dose can be made using the imaging dose values previously reported in the literature. Thengumpallil et al. measured the cone-beam dose index (CBDI) for the Elekta Symmetry protocol, with which 1320 projections were acquired with a 20 mA tube current during 200° gantry rotation; the measured CBDI was 17.6 mGy [25]. Supposing that the fractional dose for the lung SBRT is 10 Gy (median across the 16 VMAT plans in this study), the 4D CBCT imaging dose is estimated to be 0.2% of the radiation dose for treatment. The mean number of kV projections acquired for the 4D in-treatment CBCT was 617, which is approximately half of that for the 4D pretreatment CBCT in Thengumpallil et al. Therefore, the imaging dose for 4D in-treatment CBCT with a 40 mA tube current is estimated to be, on average, similar to the imaging dose reported (17.6 mGy). For the patients, to whom high MUs are delivered, the imaging dose for 4D in-treatment CBCT with a 40 mA tube current can be larger than the reported dose. For instance, in this study, the largest number of kV projections acquired was 1194 in this investigation. The corresponding imaging dose can be estimated to be almost twice as high as the reported dose: 17.6 mGy × (1194/1320) × (40 mA/20 mA) = 17.6 mGy × 1.8 = 31.7 mGy.

Furthermore, it was demonstrated how the MUs of the VMAT plan concurrently delivered affects the image quality of in-treatment CBCT. For the in-treatment CBCT, a large number of kV projections (or slow gantry rotation) was found to be highly correlated with large MUs. In other words, as the MUs increased (i.e. the gantry moved more slowly), a larger number of projections were obtained. While an improvement in the CBCT image quality is expected due to the increase in the number of projections acquired, image quality degradation could also be expected due to the increase in the MV scatter. The positive correlations found between the MUs and CNR demonstrated that the effect of the number of kV projections was dominant over that of the MV treatment beam scatter. This finding suggests that in-treatment CBCT should be acquired with an arc with higher MUs in the case of multiple-arc VMAT delivery.

The localization accuracy of a moving target was evaluated using the XSight lung tracking phantom, in which a respiratory motion of a lung target was simulated with a specific breathing condition and target size. The motion range of approximately 3 cm, which was simulated in the XSight lung tracking phantom, was relatively large compared to the lung motion ranges reported in the previous studies (see Table 1 in the report of the AAPM Task Group 76 [1]). Considering that the breathing cycle of 5 s is close to the average cycle, the evaluation of the target visibility and localization accuracy using the 4D in-treatment CBCT was performed for a fast-moving lung target. Therefore, in this sense, a similar or superior localization accuracy is expected for lung patients. However, this study is limited since the investigation was carried out for only one target size (2.5 cm). For smaller lung targets, the target localization may be more affected by the MV treatment scatter artifacts. Although it was demonstrated that the lung target in the XSight tracking phantom was localized with sub-millimeter accuracy, similar to the results in a recent study [20], target localization accuracy may be lower in patient cases.

Several studies have investigated the feasibility of reducing the MV treatment scatter effect on the in-treatment CBCT image quality [13, 14, 24, 26]. The first work to acquire MV scatter-free CBCT images was performed by Ling et al.; in this study, a control point was divided into two points, that is, a MV treatment control point and a kV imaging control point to minimize the interference between the MV and kV beams. One limitation of the proposed method is the increase in the radiation beam-on time. Another scatter correction method was suggested by van Herk et al. [24], in which kV imaging acquisition was alternated while MV treatment beams were turned on throughout a gantry rotation. In this previous work, MV scatter was estimated using kV-on and kV-off projections. However, a limitation of the method proposed by van Herk et al. was the decrease in the number of kV projections acquired, which had a negative effect on the CBCT image quality. Although a direct measurement method for MV scatter at the first treatment fraction was suggested by Boylan et al. [14], acquiring the in-treatment CBCT at the first fraction is inherently infeasible. Recently, a hardware-based scatter correction approach [26] was suggested by Ouyang et al., but it has not been implemented for clinical use. Further investigations need to be performed to establish a clinically feasible solution for MV scatter correction in in-treatment CBCT images.

## Conclusion

The image quality of 4D in-treatment CBCT was degraded by the scatter of MV FFF treatment beams used for lung SBRT. In order to cope with the image quality degradation of 4D in-treatment CBCT, based on the evaluation results, full-arc acquisition with an increased tube current and the selection of a treatment beam with higher MUs is recommended. The respiratory motion range of a lung target can be accurately calculated using 4D in-treatment CBCT as shown by the localization accuracy of the lung target in the XSight tracking phantom. These results demonstrated that 4D in-treatment CBCT with the appropriate selection of acquisition parameters can be used to monitor intrafraction variation in the lung target position during VMAT-based SBRT.

## Data Availability

All data generated or analysed during this study are included in this published article.

## Abbreviations

CBCT: Cone-beam CT
CBDI: Cone-beam dose index
CNR: Contrast-to-noise ratio
CT: Computed tomography
FFF: Flattening filter-free
MU: Monitor unit
MV: Megavoltage
ROI: Region of interest
SBRT: Stereotactic body radiation therapy
VMAT: Volumetric-modulated arc therapy
3D: Three-dimensional
4D: Four-dimensional

## References

[1] Keall PJ, Mageras GS, Balter JM, Emery RS, Forster KM, Jiang SB (2006). The management of respiratory motion in radiation oncology report of AAPM Task Group 76. Med Phys.

[2] Hoisak JDP, Sixel KE, Tirona R, Cheung PCF, Pignol JP (2004). Correlation of lung tumor motion with external surrogate indicators of respiration. Int J Radiat Oncol Biol Phys.

[3] Berbeco RI, Nishioka S, Shirato H, Chen GTY, Jiang SB (2005). Residual motion of lung tumours in gated radiotherapy with external respiratory surrogates. Phys Med Biol.

[4] Malinowski KT, McAvoy TJ, George R, Dieterich S, D’Souza WD (2012). Mitigating errors in external respiratory surrogate-based models of tumor position. Int J Radiat Oncol Biol Phys..

[5] Shimizu S, Shirato H, Ogura S, Akita-Dosaka H, Kitamura K, Nishioka T (2001). Detection of lung tumor movement in real-time tumor-tracking radiotherapy. Int J Radiat Oncol Biol Phys.

[6] Seppenwoolde Y, Shirato H, Kitamura K, Shimizu S, Van Herk M, Lebesque JV (2002). Precise and real-time measurement of 3D tumor motion in lung due to breathing and heartbeat, measured during radiotherapy. Int J Radiat Oncol Biol Phys.

[7] Li WZ, Liang ZW, Cao Y, Cao TT, Quan H, Yang ZY (2019). Estimating intrafraction tumor motion during fiducial-based liver stereotactic radiotherapy via an iterative closest point (ICP) algorithm. Radiat Oncol.

[8] Keall PJ, Aun Ng J, O’Brien R, Colvill E, Huang C-Y, Rugaard Poulsen P (2015). The first clinical treatment with kilovoltage intrafraction monitoring (KIM): a real-time image guidance method. Med Phys.

[9] Shieh C-C, Keall PJ, Kuncic Z, Huang C-Y, Feain I (2015). Markerless tumor tracking using short kilovoltage imaging arcs for lung image-guided radiotherapy. Phys Med Biol.

[10] Kim J, Park YK, Edmunds D, Oh K, Sharp GC, Winey B (2018). Kilovoltage projection streaming-based tracking application (KiPSTA): First clinical implementation during spine stereotactic radiation surgery. Adv Radiat Oncol.

[11] Nakagawa K, Yamashita H, Shiraishi K, Igaki H, Terahara A, Nakamura N (2007). Verification of in-treatment tumor position using kilovoltage cone-beam computed tomography: a preliminary study. Int J Radiat Oncol.

[12] Nakagawa K, Haga A, Shiraishi K, Yamashita H, Igaki H, Terahara A (2009). First clinical cone-beam CT imaging during volumetric modulated arc therapy. Radiother Oncol.

[13] Ling C, Zhang P, Etmektzoglou T, Star-lack J, Sun M, Shapiro E (2011). Acquisition of MV-scatter-free kilovoltage CBCT images during RapidArc^TM^ or VMAT. Radiother Oncol.

[14] Boylan CJ, Marchant TE, Stratford J, Malik J, Choudhury A, Shrimali R (2012). A megavoltage scatter correction technique for cone-beam CT images acquired during VMAT delivery. Phys Med Biol.

[15] Takahashi W, Yamashita H, Kida S, Masutani Y, Sakumi A, Ohtomo K (2013). Verification of planning target volume settings in volumetric modulated arc therapy for stereotactic body radiation therapy by using in-treatment 4-dimensional cone beam computed tomography. Int J Radiat Oncol Biol Phys.

[16] Bellec J, Arab-Ceschia F, Castelli J, Lafond C, Chajon E (2020). ITV versus mid-ventilation for treatment planning in lung SBRT: a comparison of target coverage and PTV adequacy by using in-treatment 4D cone beam CT. Radiat Oncol.

[17] Yoganathan SA, Maria Das KJ, Maria Midunvaleja K, Gowtham Raj D, Agarwal A, Velmurugan J (2015). Evaluating the image quality of cone beam CT acquired during rotational delivery. Br J Radiol.

[18] Shimohigashi Y, Doi Y, Kouno Y, Yotsuji Y, Maruyama M, Kai Y (2019). Image quality evaluation of in-treatment four-dimensional cone-beam computed tomography in volumetric-modulated arc therapy for stereotactic body radiation therapy. Phys Medica.

[19] Zijp L, Sonke J-J, van Herk M. Extraction of the respiratory signal from sequential thorax Cone-Beam X-ray images. In: Proc 14th ICCR. Seoul; 2004. p. 507–9.

[20] Liang J, Lack D, Zhou J, Liu Q, Grills I, Yan D (2019). Intrafraction 4D-cone beam CT acquired during volumetric arc radiotherapy delivery: kV parameter optimization and 4D motion accuracy for lung stereotactic body radiotherapy (SBRT) patients. J Appl Clin Med Phys.

[21] Ahmad M, Pan T (2012). Target-specific optimization of four-dimensional cone beam computed tomography. Med Phys.

[22] Yoganathan SA, Das KJM, Ali SM, Agarwal A, Mishra SP, Kumar S (2016). Evaluating the four-dimensional cone beam computed tomography with varying gantry rotation speed. Br J Radiol.

[23] Shimohigashi Y, Araki F, Maruyama M, Yonemura K, Nakaguchi Y, Kai Y (2018). Image quality of four-dimensional cone-beam computed tomography obtained at various gantry rotation speeds for liver stereotactic body radiation therapy with fiducial markers. Phys Medica.

[24] van Herk M, Ploeger L, Sonke J-J (2011). A novel method for megavoltage scatter correction in cone-beam CT acquired concurrent with rotational irradiation. Radiother Oncol.

[25] Thengumpallil S, Smith K, Monnin P, Bourhis J, Bochud F, Moeckli R (2016). Difference in performance between 3D and 4D CBCT for lung imaging: a dose and image quality analysis. J Appl Clin Med Phys.

[26] Ouyang L, Lee HP, Wang J (2015). A moving blocker-based strategy for simultaneous megavoltage and kilovoltage scatter correction in cone-beam computed tomography image acquired during volumetric modulated arc therapy. Radiother Oncol.

